# Regional and temporal progression of atrial remodeling in angiotensin II mediated atrial fibrillation

**DOI:** 10.3389/fphys.2022.1021807

**Published:** 2022-11-01

**Authors:** Hailey J. Jansen, Megan D. McRae, Martin Mackasey, Robert A. Rose

**Affiliations:** Libin Cardiovascular Institute, Department of Cardiac Sciences, Department of Physiology and Pharmacology, Cumming School of Medicine, University of Calgary, Calgary, AB, Canada

**Keywords:** atrial fibrillation, action potential, ion channels, fibrosis, atrial remodeling

## Abstract

Atrial fibrillation (AF) is associated with electrical and structural remodeling in the atria; however, the regional and temporal progression of atrial remodeling is incompletely understood. The objective of this study was to investigate the regional and temporal progression of atrial remodeling leading to changes in AF susceptibility in angiotensin II (Ang II) mediated hypertension. Mice were infused with Ang II for 3, 10 or 21 days. AF susceptibility and atrial electrophysiology were studied *in vivo* using intracardiac electrophysiology. Right and left atrial myocyte electrophysiology was studied using patch-clamping. Atrial fibrosis was assessed histologically. P wave duration and atrial effective refractory period increased progressively from 3 to 21 days of Ang II. AF susceptibility tended to be increased at 10 days of Ang II and was elevated at 21 days of Ang II. Left, but not right, atrial AP upstroke velocity and Na^+^ current were reduced at 10 and 21 days of Ang II. Left atrial action potential (AP) duration increased progressively from 3 to 21 days of Ang II due to reductions in repolarizing K^+^ current. Right atrial AP prolongation was increased only after 21 days of Ang II. Left and right atrial fibrosis developed progressively from 3 to 21 days, but increases were larger in the left atrium. In conclusion, Ang II mediated atrial electrical and structural remodeling develop earlier and more extensively in the left atrium compared to the right atrium, providing insight into how atrial remodeling leads to enhanced AF susceptibility in Ang II mediated hypertension.

## Introduction

Atrial fibrillation (AF) is the most commonly encountered sustained cardiac arrhythmia and is associated with a reduced quality of life and an increased risk of stroke, heart failure, and mortality (Heijman et al., 2014; Jansen et al., 2020; Kornej et al., 2020). Hypertension, which affects over 1.3 billion patients worldwide (Bloch, 2016), is an important risk factor for AF (Gumprecht et al., 2019; Jansen et al., 2020; Kornej et al., 2020). It has been estimated that 60–80% of patients with AF are also diagnosed with high blood pressure (Verdecchia et al., 2018). Furthermore, the incidence of AF increases with a longer duration of hypertension (Verdecchia et al., 2018; Kim et al., 2019). Hypertension is associated with activation of the renin-angiotensin system and elevated Angiotensin II (Ang II) levels are associated with the development of AF (Khatib et al., 2013; Heijman et al., 2014; Jansen et al., 2020; Nattel et al., 2020; Aguilar et al., 2021). Consistent with these clinical findings, numerous studies have demonstrated that Ang II infusion in mice generates a model of increased AF susceptibility (Fukui et al., 2013; Purohit et al., 2013; Jansen et al., 2018; Li et al., 2018; Jansen et al., 2019). Ang II mediates its effects in part by binding to the Ang II type 1 receptor (AT1R) (Forrester et al., 2018).

AF, including in association with Ang II infusion, is associated with atrial electrical and structural remodeling (Schotten et al., 2011; Jansen et al., 2020; Nattel et al., 2020; Aguilar et al., 2021). The action potential (AP) is a critical determinant of atrial electrophysiology and alterations in atrial AP morphology can contribute to the initiation and/or maintenance of AF (Jansen et al., 2020; Nattel et al., 2020). The sodium current (I_Na_, carried by Na_v_1.5 channels) is responsible for the AP upstroke and plays a key role in electrical impulse propagation (Kleber and Rudy, 2004; Bartos et al., 2015). Changes in I_Na_ and AP upstroke velocity (V_max_) can affect atrial conduction velocity and the wavelength of re-entry, creating a substrate for AF maintenance (Jansen et al., 2020; Nattel et al., 2020). AP repolarization is influenced by several repolarizing potassium currents that include the transient outward potassium current (I_to_, carried by K_V_4.2/4.3 channels), the ultra rapid delayed rectifier potassium current (I_Kur_, carried by K_v_1.5 channels), and a steady state potassium current (I_Kss_, carried by K_V_2.1 channels) (Nerbonne and Kass, 2005; Bartos et al., 2015; Jansen et al., 2020). In some species, the rapid delayed rectifier potassium current (I_Kr_, carried by K_V_11.1) and the slowly activated delayed rectifier potassium current (I_Ks_, carried by K_V_7.1) also contribute to AP repolarization (Nerbonne and Kass, 2005). Alterations in these ionic currents can prolong the AP and promote conduction disturbances, re-entry, or the development of early afterdepolarizations that can serve as triggers for AF (Jansen et al., 2020; Nattel et al., 2020).

AF is also associated with structural remodelling due to fibrosis (Jalife and Kaur, 2015; Nattel, 2017; Jansen et al., 2020). Atrial fibrosis can develop in association with increased interstitial collagen production and deposition as well as alterations in remodeling of the extracellular matrix by matrix metalloproteinases (MMPs) and tissue inhibitors of metalloproteinases (TIMPs) (Nattel, 2017; Jansen et al., 2020). Increases in interstitial collagen can disrupt atrial myocyte connectivity leading to slow atrial conduction velocity and conduction block thereby promoting electrical re-entry. These structural changes can favor the maintenance of AF.

We have recently demonstrated that chronic Ang II infusion is associated with distinct patterns of electrical and structural remodelling in the right and left atria (Jansen et al., 2018; Jansen et al., 2019). Specifically, Ang II infusion caused a reduction in left atrial AP V_max_ in association with a reduction in left atrial I_Na_. Ang II infusion also resulted in increases in atrial AP duration (APD) that occurred in association with reductions in repolarizing I_K_. Furthermore, interstitial collagen levels were increased in the right and left atria. These alterations in electrical and structural remodelling were associated with an increased susceptibility to AF in Ang II infused mice.

While electrical and structural remodeling are clearly associated with the Ang II-mediated increase in susceptibility to AF, the regional and temporal effects of Ang II infusion on atrial remodeling remains incompletely understood. Accordingly, the goal of this study was to investigate the time course of the progression of electrical and structural remodelling in the right and left atria, and the resulting susceptibility to AF in Ang II infused mice. Our data demonstrate that electrical and structural remodeling develop earlier and progress more rapidly in the left atrium compared to the right atrium. These data provide important insight into the patterns of atrial remodeling that lead to increased susceptibility to AF.

## Materials and methods

### Mice

This study used male wildtype C57bl/6 mice between the ages of 10–15 weeks. Mice were implanted with subcutaneous osmotic minipumps (Alzet) for infusion of Ang II (2.5 mg/kg/day) for 3, 10, or 21 days using procedures described previously (Jansen et al., 2018; Jansen et al., 2019). Control mice were infused with saline for 21 days. All experimental procedures were approved by the University of Calgary Animal Care and Use Committee and were in accordance with the guidelines of the Canadian Council on Animal Care.

### Blood pressure

Blood pressure was measured in conscious, restrained mice by tail-cuff plethysmography (IITC Life Sci). Measurements were taken in the same mice at baseline (before osmotic pump implantation) and following 3, 10, and 21 days of Ang II infusion.

### *In vivo* electrophysiology and arrhythmia studies

Surface ECGs were recorded in anaesthetized mice (2% isoflurane inhalation) using 30-gauge subdermal needle electrodes (Grass Technologies). A 1.2 French octopolar electrophysiology catheter (Transonic) was advanced into the right heart via the jugular vein as we have previously described (Jansen et al., 2018; Mackasey et al., 2018; Jansen et al., 2019). Correct catheter placement was achieved by a strong atrial signal in the proximal lead and a ventricular signal in the distal lead. Rapid burst pacing of the right atrium was used to assess the inducibility of AF. We used 5 high frequency burst pacing protocols to induce mice into AF and mice were allowed to stabilize for 2 min between each stimulation. The first protocol was a 2 s burst applied at a cycle length of 40 ms with a pulse duration of 5 ms. The second 2 s burst was applied at a cycle length of 20 ms with a pulse duration of 5 ms. The third 2 s burst was delivered at a cycle length of 20 ms and a pulse duration of 10 ms. These protocols were followed by two 100 beat protocols delivered at either 25 or 20 ms pulse duration. AF was defined as a rapid and irregular atrial rhythm as demonstrated by a fibrillatory baseline in the ECG in association with irregular RR intervals that lasted at least 1 s on the surface ECG. All ECG data were acquired on a Gould ACQ-7700 amplifier and Ponemah Physiology Platform software (Data Sciences International).

AERP was measured using an S1-S2 protocol that consisted of 8 beats delivered at a fixed cycle length of 100 ms (S1) followed by a single beat (S2) delivered at progressively shorter cycle lengths. Body temperature was measured using a rectal probe and maintained at 37°C using a heating pad.

### Isolation of mouse atrial myocytes

Right and left atrial myocytes were isolated as previously described (Egom et al., 2015; Jansen et al., 2018; Jansen and Rose, 2019). Briefly, mice were anaesthetized using isoflurane inhalation and then sacrificed by cervical dislocation. The heart was excised into Tyrode’s solution consisting of (in mM): 140 NaCl, 5.4 KCl, 1.2 KH_2_PO_4_, 1.0 MgCl_2_, 1.8 CaCl_2_, 5.55 glucose, and 5 HEPES, with pH adjusted to 7.4 with NaOH. Heparin was added to the Tyrode’s solution to prevent blood clotting and all solutions were warmed to 35°C unless otherwise stated. The right or left atrial appendage was dissected from the heart, cut into strips and allowed to equilibrate for 5 min in a ‘low Ca^2+,^ Mg^−2+^ free’ solution containing (in mM): 140 NaCl, 5.4 KCl, 1.2 KH_2_PO_4_, 0.2 CaCl_2_, 50 taurine, 18.5 glucose, 5 HEPES and 1 mg/ml bovine serum albumin (BSA), with pH adjusted to 6.9 with NaOH. Next, atrial tissue strips were digested for 30 min in 5 ml of the ‘low Ca^2+,^ Mg^−2+^ free’ solution containing 1064 units collagenase (type II, Worthington Biochemical Corporation), 9 units elastase (Worthington Biochemical Corporation), and 62.5 μl of 1 mg/100 μl solution of protease from *Streptomyces griseus* (type XIV, Sigma-Aldrich). Tissue strips were intermittently swirled during enzymatic digestion. Next, digested tissue strips were washed 3 times in 2.5 ml of modified Kraft-Brühe (KB) solution containing (in mM): 100 potassium glutamate, 10 potassium aspartate, 25 KCl, 10 KH_2_PO_4_, 2 MgSO_4_, 20 taurine, 5 creatine, 0.5 EGTA, 20 glucose, 5 HEPES, and 0.1% BSA, with pH adjusted to 7.2 with KOH. Following a 5 min incubation these tissue strips were mechanically dissociated using a wide-bore pipette. This procedure successfully yields individual right or left atrial myocytes that were stored at room temperature in KB solution and used for electrophysiology experiments within 6 h of isolation.

### Patch-clamping of atrial myocytes

Patch-clamping studies were performed in isolated right and left atrial myocytes to record stimulated APs and ionic currents in the whole cell configuration as we have done previously (Jansen et al., 2018; Jansen et al., 2019). Stimulated action potentials (APs) were recorded in isolated right or left atrial myocytes using the whole cell configuration of the patch-clamp technique. To record APs, atrial myocytes were superfused with a normal Tyrode’s solution at room temperature containing (in mM): 140 NaCl, 5 KCl, 1 MgCl_2_, 1 CaCl_2_, 10 HEPES, and 5 glucose, with pH adjusted to 7.4 with NaOH. The pipette filling solution contained (in mM): 135 KCl, 0.1 CaCl2, 1 MgCl_2_, 5 NaCl, 10 EGTA, 4 Mg-ATP, 6.6 Na-phosphocreatine, 0.3 Na-GTP and 10 HEPES, with pH adjusted to 7.2 with KOH. The resting membrane potential was corrected for a liquid junction potential of 4.6 mV. I_Na_ was recorded using a voltage clamp protocol that consisted of a series of 50 ms voltage clamp steps from -100 to +50 mV in 10 mV increments from a holding potential of -120 mV. To record I_Na_, atrial myocytes were superfused with a modified Tyrode’s solution containing (in mM): 130 CsCl, 5 NaCl, 5.4 TEA-Cl, 1 MgCl_2_, 1 CaCl_2_, 10 HEPES, 5.5 glucose and adjusted to pH 7.4 with CsOH and supplemented with 10 μM nifedipine to block I_Ca,L_. The pipette solution contained (in mM): 5 NaCl, 130 CsCl, 1 MgCl_2_, 0.2 CaCl_2_, 10 HEPES, 5 BAPTA, 5 Mg-ATP, 0.3 Na-GTP and adjusted to pH 7.2 with CsOH. I_Na_ activation kinetics were determined by calculating the chord conductance (G) with the equation G=I(V_m_-E_rev_) where V_m_ represents the depolarizing potential and E_rev_ is the reversal potential obtained from the current-voltage relationships of I_Na_ for each cell. Maximum conductance (G_max_) and V_1/2_ activation (V_1/2(act)_) were calculated using the following function: G=[(V_m_- V_rev_)][G_max_][-1/[(1+exp ((V_m_-V_1/2_)/k))+1]].

Potassium currents (I_K_) were recorded in the whole cell configuration of the patch-clamp technique using the same Tyrode’s and pipette solutions used to record APs. To record total I_K_ cells were recorded using a series of 500 ms voltage clamp steps between the membrane potentials of -120 to +80 mV from a holding potential of -80 mV. To record I_k_ with a pre-pulse to inactivate I_to_, cells were given a 200 ms pre-pulse to -40 mV immediately followed by 500 ms voltage clamp steps from -120 to +80 mV from a holding potential of -80 mV. I_k_ was measured as the peak current at each membrane potential and I_to_ was measured as the difference current with and without the inactivating pre-pulse (Jansen et al., 2018; Jansen et al., 2019).

Micropipettes were pulled from borosilicate glass (with filament, 1.5 mm OD, 0.75 mm ID, Sutter Instrument Company) using a Flaming/Brown pipette puller (model p-87, Sutter Instrument Company). The resistance of these pipettes was 4–8 MΩ when filled with pipette solution. Micropipettes were positioned using a micromanipulator (Burleigh PCS-5000 system) mounted on the stage of an inverted microscope (Olympus IX71). Seal resistances were 2–15 GΩ following sarcolemma rupture and series resistance was compensated to 85% using an Axopatch 200B amplifier (Molecular Devices). Data were digitized using a Digidata 1440 and pCLAMP 10 software (Molecular Devices) then stored on a computer for analysis. All patch-clamp studies were conducted at room temperature and currents were normalized to the cell capacitance.

### Collagen staining

Interstitial collagen was assessed using picrosirius red and fast green staining of sections through the right and left atrial appendages as we have previously described previously (Jansen et al., 2018; Jansen et al., 2019; Bohne et al., 2021). Atrial tissue was fixed for 3 days in 10% neutral buffered formalin solution (Sigma-Aldrich). Fixed tissue was then processed and embedded in paraffin wax before 5 μm sections were adhered to glass slides. Next slides were deparaffinized using three 5 min incubations in xylene followed by a series of 5 min rehydration steps consisting of two washes in 100% ethanol, two washes in 95% ethanol, one wash in 75% ethanol followed by two 5 min washes in water. Next slides were stained for 1 h in 0.1% picrosirius red (to stain collagen) with 0.2% fast green FCF (to counterstain for myocardial tissue) for 1.5 h at room temperature. This was immediately followed by one wash in water, 2 washes in 75% ethanol, 1 wash in 95% ethanol, 2 washes in 100% ethanol, and 3 washes in xylene. Slides were then mounted with mounting media and images taken at 40x magnification. The level of interstitial fibrosis was quantified using ImageJ software as the percentage of red (i.e. collagen) to green (i.e. muscle) tissue from at least 8 regions from each sample.

### Quantitative PCR

Quantitative gene expression was measured in the right and left atria. Intron-spanning primers for *agtr1* (encodes Ang II type 1 receptor) and hypoxanthine phosphoribosyltransferase (*hprt1*; reference gene) were used. Primer sequences are listed in Table 1.

**TABLE 1:** qPCR primers.

Gene	Gene product	Primer sequence (5’→ 3′)	Amplicon length (bp)
*agtr1*	AT1R	Forward: CCC​TGG​CTG​ACT​TAT​GCT​TT	92
		Reverse: ACA​TAG​GTG​ATT​GCC​GAA​GG	
*hprt1*	Hypoxanthine phosphoribosyltranferase 1	Forward: GCA​GGT​CAG​CAA​AGA​ACT​TAT​AGC​C	123
		Reverse: CTC​ATG​GAC​TGA​TTA​TGG​ACA​GGA​C	

Total RNA was isolated from right or left atrial appendages using a PureZOL^TM^ RNA Isolation Reagent and the Aurum^TM^ Total RNA Fatty and Fibrous Tissue Kit (Bio-Rad Laboratories) as per kit instructions. RNA samples were eluted from the spin column in 40 µl elution buffer. RNA yield and purity were assessed using a Nanodrop. All samples had a A_260_/A_280_ of over 2.0 and therefore were free of DNA contamination. Next, cDNA (5 ng/µl) was synthesized using the iScript^TM^ cDNA Synthesis Kit (Bio-Rad Laboratories). Reactions were performed in a Bio-Rad MyCycler thermal cycler using the following protocol: 5 min of priming at 25°C followed by reverse transcription for 30 min at 42°C then 5 min at 85°C to inactivate reverse transcriptase.

All qPCR reactions were run in duplicate in 10 µl reactions that contained the following: 4 µl sample cDNA, 5.6 µl GoTaq^®^ qPCR Master Mix (Promega), and 0.4 µl primers. Primers were reconstituted to a final concentration of 100 µM with nuclease free water and stored at -20°C until use. Primers were diluted to 10 µM for qPCR reactions. RT-qPCR reactions were performed using the CFX386 Touch^TM^ Real-Time PCR Detection System (Bio-Rad) using the following protocol: Taq polymerase was activated for 2 min at 95°C followed by 39 cycles of denaturing for 15 s at 95°C, annealing for 30 s at 60°C, and extension for 30s at 72°C. This was followed by melt curve analysis from 65 to 95°C in 0.5°C increments. Data were analyzed using the 2^−ΔΔCT^ method using the CFX Manager Software version 3.1 (Bio-Rad).

### Statistical analysis

All data are presented as mean ± SEM. Data were analyzed using Fisher’s exact test, Student’s *t*-test, one way analysis of variance with a Holm-Sidak *post hoc* test, or two-way repeated measures analysis of variance with a Holm-Sidak *post hoc* test as indicated in the figure legends. *p* < 0.05 was considered significant.

## Results

### Time course of Ang II effects on atrial fibrillation and atrial electrophysiology

**FIGURE 1:**
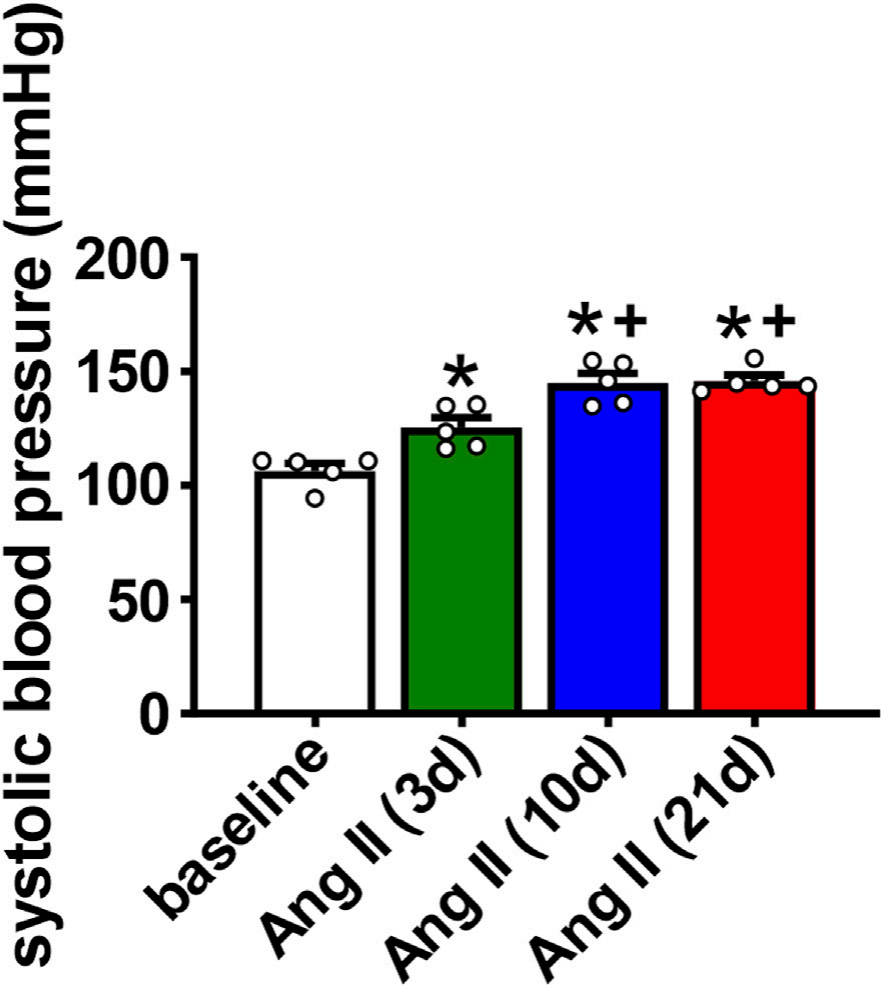
Time course of the effects of Ang II on systolic blood pressure. Systolic pressure was measured at baseline, 3 days, 10 days and 21 days of Ang II infusion. *p < 0.05 vs baseline; ^+^ *p* < 0.05 vs Ang II (3 days) by one-way repeated measures ANOVA with a Holm-Sidak posthoc test; *n*=5 mice per group.

Wildtype mice were infused with Ang II for 3, 10 or 21 days to determine the time course of atrial arrhythmogenesis as well as atrial electrical and structural remodeling. Over this time frame Ang II elicited a progressive increase in systolic blood pressure that was evident at 3 days of Ang II infusion and maximally elevated by 10 days of Ang II treatment (Figure 1). No further increase in systolic blood pressure was seen at the 21 days time point.

**FIGURE 2:**
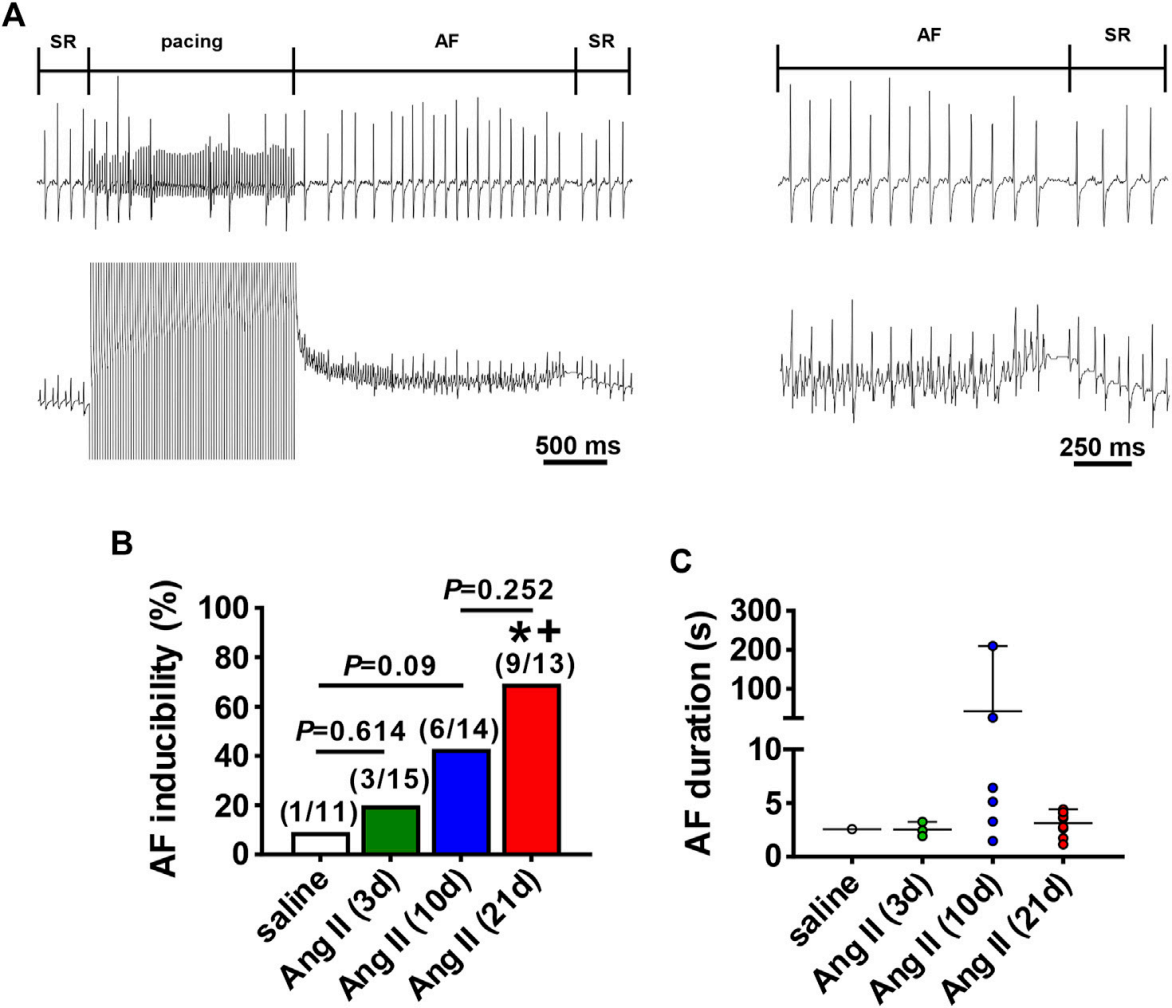
Susceptibility to atrial fibrillation after Ang II infusion. **(A)** Representative surface (top) and intracardiac (bottom) ECGs illustrating the induction of AF after burst pacing in the right atrium in a mouse infused with Ang II. The recordings on the right are magnified regions of the recordings on the left showing the ECGs during AF and the spontaneous reversion back to sinus rhythm (SR). **(B)** Inducibility of AF in mice infused with saline for 21 days or Ang II for 3, 10 and 21 days. Numbers in parentheses indicate the number of mice induced into AF. *p < 0.05 vs saline; ^+^ *p* < 0.05 vs Ang II (3 days) by Fisher’s exact test. **(C)** Duration of AF in the mice that were induced into AF as shown in panel B.

AF susceptibility was investigated using burst pacing in anesthetized mice (Figure 2A). There was no difference in AF inducibility after 3 days of Ang II infusion when compared to saline infused control mice (saline was infused for 21 days). After 10 days of Ang II infusion there was a trend (*p*=0.09) toward increased AF inducibility. By 21 days of Ang II infusion AF inducibility was increased compared to saline controls as well as 3 days of Ang II infusion (Figure 2B). In the majority of cases, AF lasted less than 10 s before reverting back to sinus rhythm although there were some cases of longer lasting AF in the mice treated with Ang II for 10 days.

**FIGURE 3:**
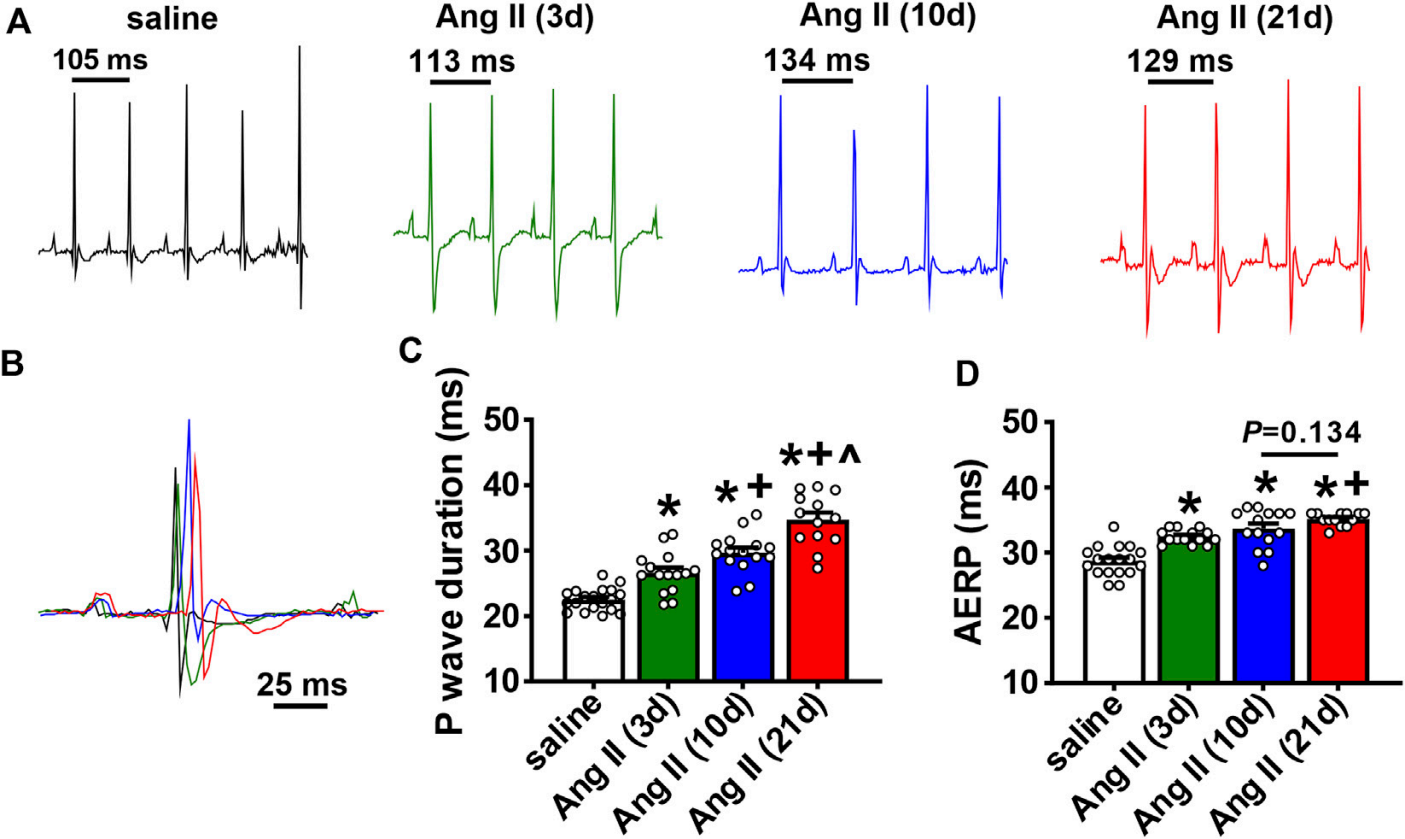
Time course of the effects of Ang II on atrial electrophysiology **(A)** Representative ECGs in mice infused with saline for 21 days or Ang II for 3, 10 and 21 days. **(B)** Overlay of ECGs showing a single beat in saline infused mice and mice infused with Ang II for 3, 10 and 21 days. Colors are as shown in panel **(A) (C)** Summary of P wave duration in mice infused with saline or Ang II for 3, 10 and 21 days. **(D)** Summary of AERP in mice infused with saline or Ang II for 3, 10 and 21 days. For panels **(C and D)** *p < 0.05 vs saline, ^+^ *p* < 0.05 vs Ang II (3 days), ^^^ *p* < 0.05 vs Ang II (10 days) by one-way ANOVA with a Holm-Sidak posthoc test; *n*=19 mice for saline, 15 for Ang II (3 days), 15 for Ang II (10 days) and 13 for Ang II (21 days).

The timing of the effects of Ang II on atrial electrophysiology were further studied using ECG analysis and programmed electrical stimulation in anesthetized mice (Figure 3). Representative ECGs (Figures 3A,B) and summary data (Figure 3C) demonstrate that P wave duration began to increase by 3 days of Ang II infusion and became progressively longer up to 21 days of Ang II infusion. AERP was also prolonged after 3 and 10 days of Ang II infusion and further increased at 21 days of Ang II infusion compared to saline controls (Figure 3D).

### Time course of Ang II effects on atrial myocyte electrophysiology

**FIGURE 4:**
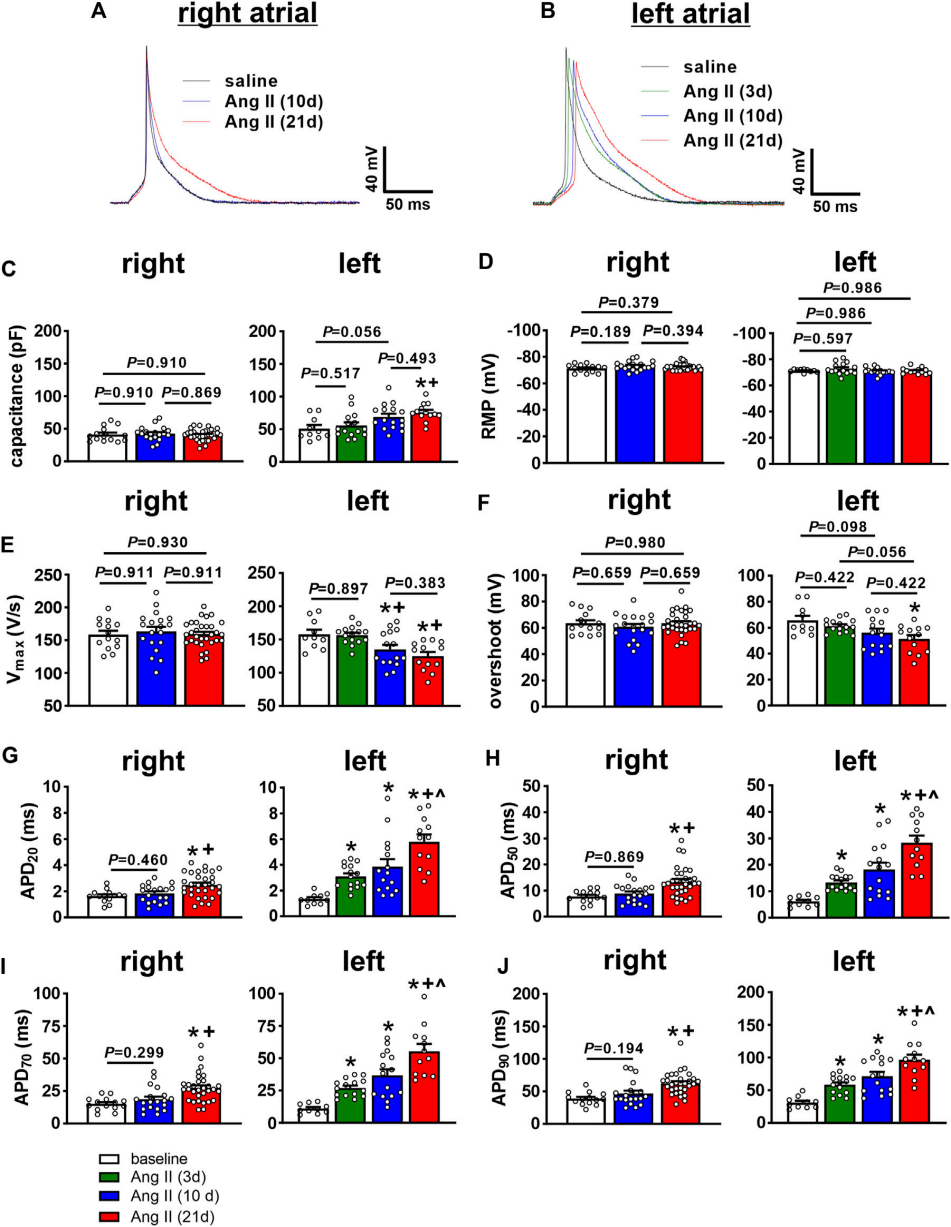
Time course of the effects of Ang II on right and left atrial AP morphology. **(A)** Representative APs in isolated right atrial myocytes from mice infused with saline for 21 days or Ang II for 10 and 21 days. **(B)** Representative APs in isolated left atrial myocytes from mice infused with saline for 21 days or Ang II for 3, 10 and 21 days. **(C–J)** Summary of cell capacitance **(C)**, resting membrane potential (RMP, **(D)**, AP upstroke velocity (V_max_, **(E)**, AP overshoot **(F)**, AP duration at 20% repolarization (APD_20_, **(G)**, APD_50_ **(H)**, APD_70_ **(I)** and APD_90_ **(J)** in right and left atrial myocytes from mice infused with saline or Ang II for the specified durations. *p < 0.05 vs saline, ^+^ *p* < 0.05 vs Ang II (3 days), ^^^ *p* < 0.05 vs Ang II (10 days) by one-way ANOVA with a Holm-Sidak posthoc test. For right atrial myocytes, *n*=14 for saline, 19 for Ang II (10 days) and 31 for Ang II (21 days). For left atrial myocytes, *n*=10 for saline, 15 for Ang II (3 days), 16 for Ang II (10 days) and 13 for Ang II (21 days).

To investigate the basis for how Ang II leads to pro-arrhythmic atrial remodeling over time, AP morphology was measured in isolated right and left atrial myocytes (Figures 4A,B) at different time points after the initiation of Ang II infusion. Cell capacitance was not altered by Ang II at any time point in right atrial myocytes (Figure 4C). In contrast, left atrial myocytes showed a trend (*p*=0.056) towards increased cell capacitance after 10 days of Ang II and a significant increase in cell capacitance after 21 days of Ang II (Figure 4C). These data indicate that left, but not right atrial myocytes develop hypertrophy in association with Ang II infusion.

There were no differences in resting membrane potential (RMP) in right or left atrial myocytes after Ang II infusion (Figure 4D). In right atrial myocytes, there were also no differences in AP upstroke velocity (V_max_, Figure 4E) or AP overshoot (Figure 4F) at any time point up to 21 days of Ang II infusion. Right atrial myocytes were not studied at 3 days of Ang II infusion due to the absence of effects at 10 days of Ang II. In left atrial myocytes, AP V_max_ was not different at 3 days of Ang II, but was reduced to similar levels at 10 and 21 days of Ang II infusion (Figure 4E). Consistent with these V_max_ changes, AP overshoot was also reduced in left atrial myocytes at 10 and 21 days of Ang II infusion (Figure 4F). AP duration was measured at 20, 50, 70 and 90% repolarization. In right atrial myocytes, APD was prolonged at each of these time points in repolarization at 21 days, but not at 10 days of Ang II infusion (Figures 4G–J). Once again, right atrial myocyte APD was not studied at 3 days of Ang II due to the absence of alterations at 10 days of Ang II. In contrast, APD_20_, APD_50_, APD_70_ and APD_90_ all increased progressively in left atrial myocytes following Ang II infusion for 3, 10 and 21 days (Figures 4G,H).

**FIGURE 5:**
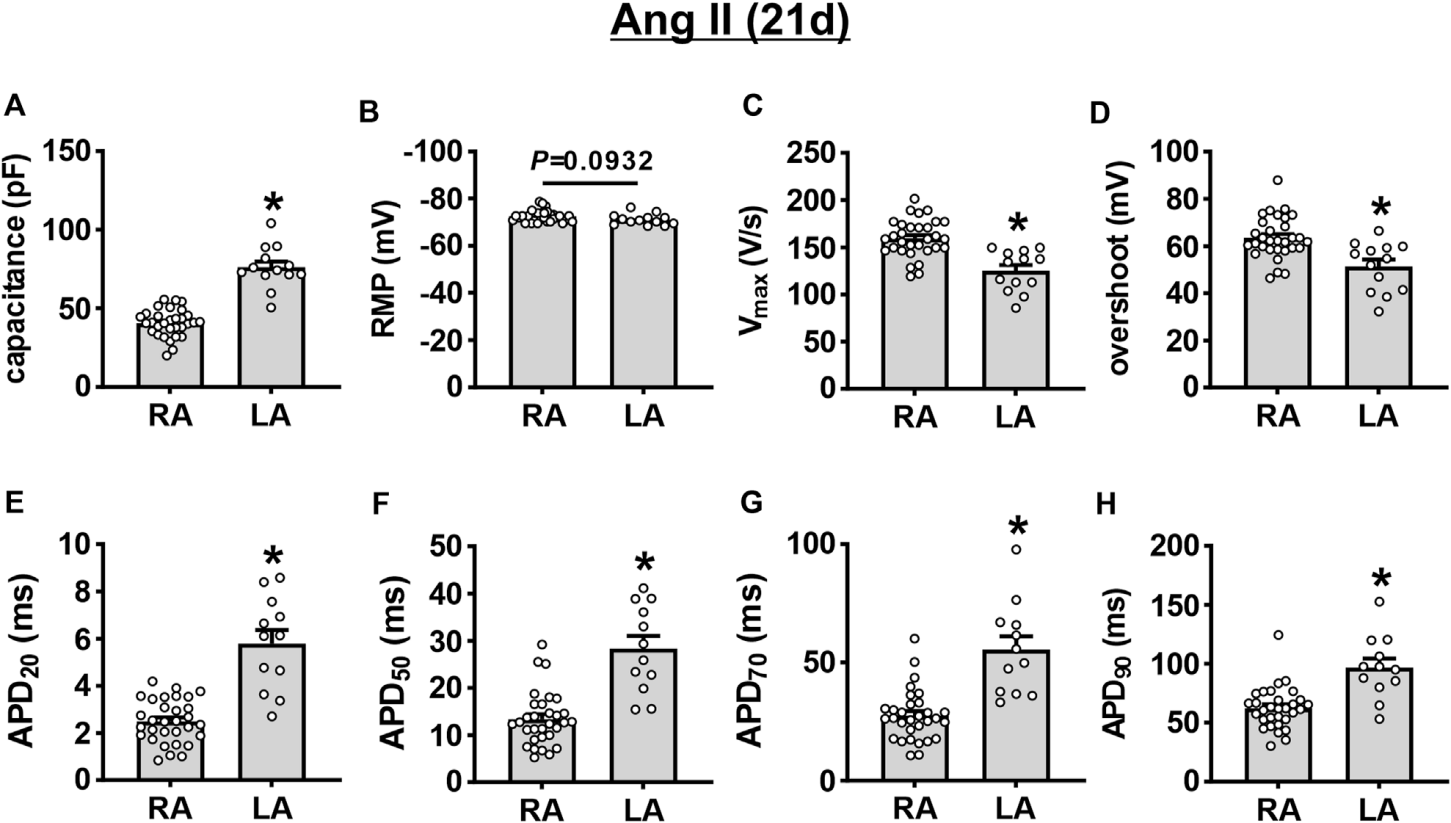
Comparison of action potential morphology in left and right atrial myocytes after 21 days of Ang II infusion. **(A–H)** Summary of cell capacitance **(A)**, RMP **(B)**, V_max_ **(C)**, overshoot **(D)**, APD_20_ **(E)**, APD_50_ **(F)**, APD_70_ **(G)**, and APD_90_ **(H)** in mouse right (RA) and left (LA) atrial myocytes at 21 days of Ang II. *p < 0.05 vs RA by Student’s *t*-test; *n*=31 myocytes for RA and 13 myocytes for LA.

AP morphology was compared between right and left atrial myocytes after 21 days of Ang II infusion (Figures 5A–H). These data demonstrate that cell capacitance was larger in left atrial myocytes compared to right atrial myocytes after 21 days of Ang II. V_max_ and overshoot were lower in left atrial myocytes compared to right atrial myocytes, while AP duration was longer throughout repolarization in left atrial myocytes after 21 days of Ang II infusion.

**FIGURE 6:**
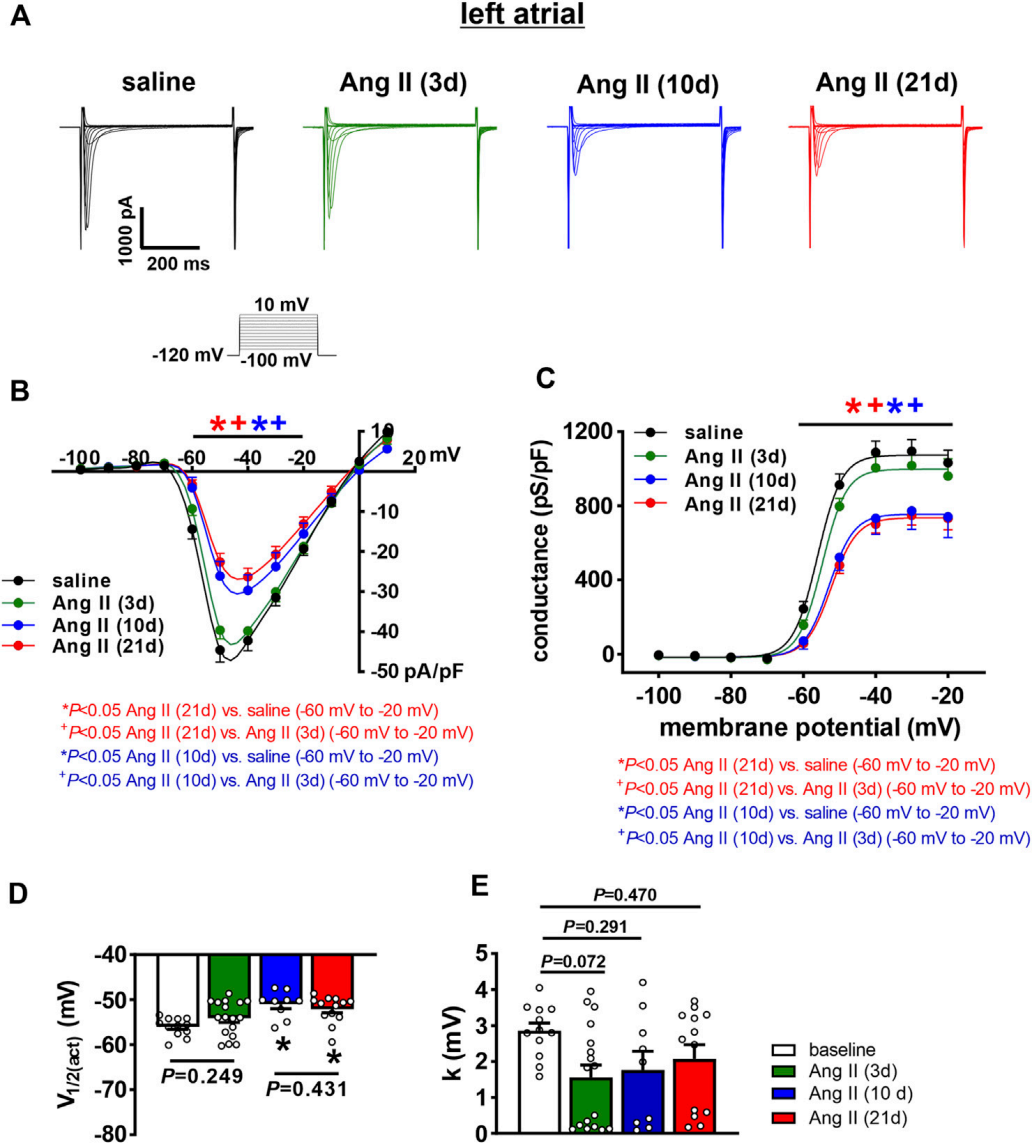
Time course of the effects of Ang II on left atrial sodium current. **(A)** Representative I_Na_ recordings in left atrial myocytes from mice infused with saline for 21 days or Ang II for 3, 10 and 21 days. I_Na_ voltage clamp protocol shown below recordings. **(B)** I_Na_ IV curves for left atrial myocytes isolated from mice infused with saline or Ang II for 3, 10, and 21 days. **(C)** I_Na_ steady-state activation curves for left atrial myocytes isolated from mice infused with saline or Ang II for 3, 10 and 21 days. **(D)** Summary of I_Na_ V_1/2(act)_ for left atrial myocytes isolated from mice infused with saline or Ang II for 3, 10 and 21 days. **(E)** Summary in I_Na_ slope factor *(k)* for left atrial myocytes isolated from mice infused with saline or Ang II for 3, 10 and 21 days. For panels **(B and C)** *p < 0.05 vs saline, ^+^ *p* < 0.05 vs Ang II (3 days) at indicated membrane potentials by two-way repeated measures ANOVA with a Holm-Sidak posthoc test. For panels **(D and E)** *p < 0.05 vs saline by one-way ANOVA with a Holm-Sidak posthoc test; *n*=12 myocytes for saline, 17 for Ang II (3 days), 9 for Ang II (10 days) and 13 for Ang II (21 days).

I_Na_ was measured only in left atrial myocytes because V_max_ was reduced in left, but not right atrial myocytes following 10 and 21 days of Ang II infusion. In left atrial myocytes, I_Na_ density was not altered at 3 days of Ang II infusion; however, I_Na_ was reduced to similar levels at 10 and 21 days of Ang II infusion (Figures 6A,B). Consistent with this, I_Na_ steady-state activation curves demonstrate that I_Na_ G_max_ was reduced at 10 and 21 days of Ang II, but not at 3 days of Ang II (Figure 6C). Analysis of the voltage dependence of I_Na_ activation demonstrates that the V_1/2(act)_ was shifted to more negative values at 10 and 21 days of Ang II, but was unaltered at 3 days of Ang II infusion (Figure 6D). There were no significant differences in the slope factor (k) of the I_Na_ activation curve between groups (Figure 6E).

**FIGURE 7:**
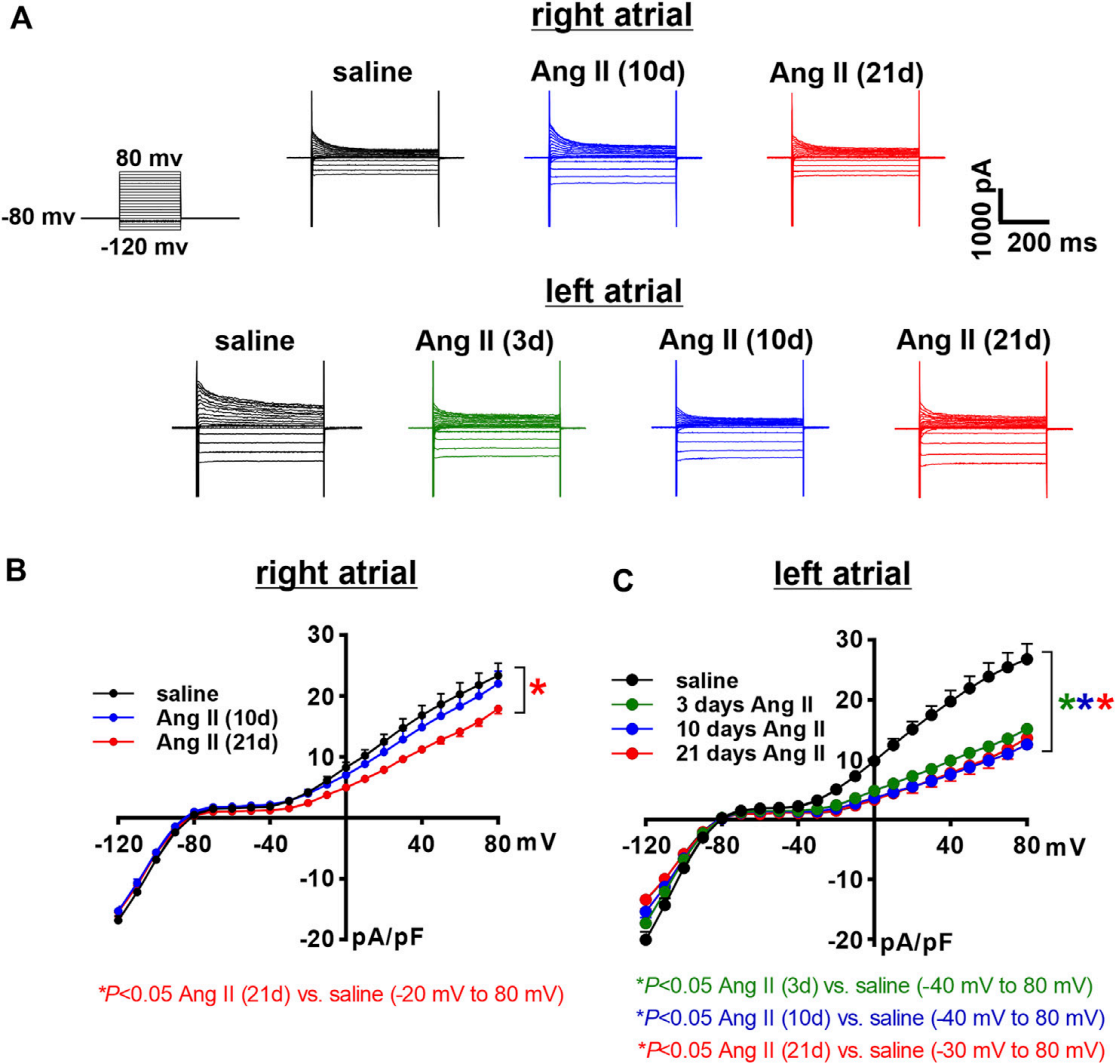
Time course of the effects of Ang II on total right and left atrial potassium currents. **(A)** Representative total I_K_ recordings in right and left atrial myocytes isolated from mice infused with saline for 21 days or Ang II for 3, 10 and 21 days. Voltage clamp protocol shown beside recordings. **(B)** Summary I_K_ IV curves measured at the peak of the I_K_ recordings for right atrial myocytes isolated from mice infused with saline or Ang II for 10 and 21 days. *p < 0.05 vs saline, ^+^ *p* < 0.05 vs Ang II (10 days) by two-way repeated measures ANOVA with a Holm-Sidak posthoc test. **(C)** Summary I_K_ IV curves measured at the peak of the I_K_ recordings for left atrial myocytes isolated from mice infused with saline or Ang II for 3, 10 and 21 days. *p < 0.05 vs saline at indicated membrane potentials by two-way repeated measures ANOVA with a Holm-Sidak posthoc test. For right atrial myocytes *n*=12 for saline, 14 for Ang II (10 days) and 21 for Ang II (21 days). For left atrial myocytes *n*=11 for saline, 15 for Ang II (3 days), 17 for Ang II (10 days) and 12 for Ang II (21 days).

**FIGURE 8:**
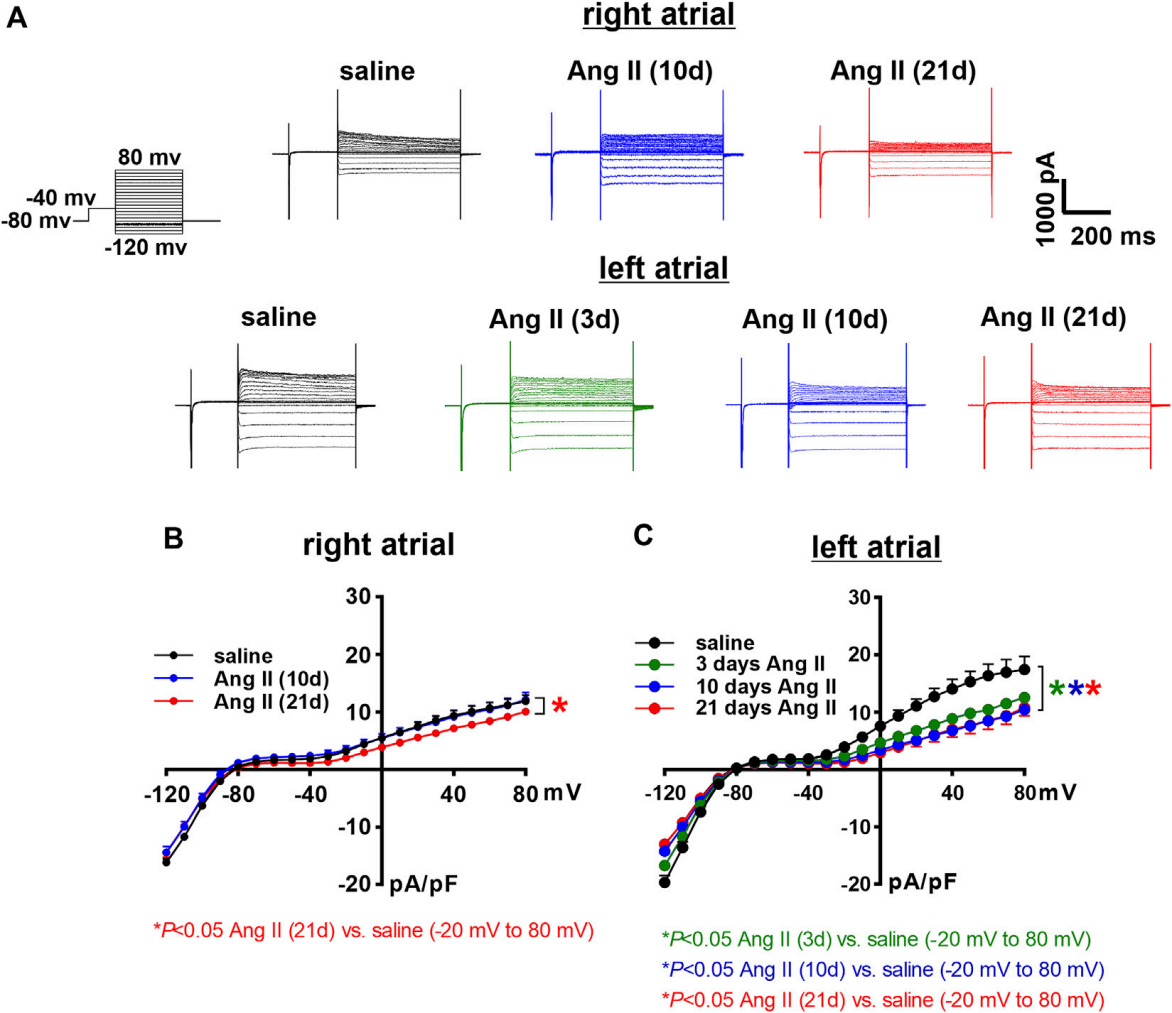
Time course of the effects of Ang II on right and left atrial potassium currents after an inactivating pre-pulse. **(A)** Representative I_K_ recordings following a pre-pulse to inactivate I_to_ in right and left atrial myocytes isolated from mice infused with saline for 21 days or Ang II for 3, 10 and 21 days. Voltage clamp protocol shown beside recordings. **(B)** Summary I_K_ IV curves, measured at the peak of the I_K_ recordings following a pre-pulse, for right atrial myocytes isolated from mice infused with saline or Ang II for 10 and 21 days. **(C)** Summary I_K_ IV curves, measured at the peak of the I_K_ recordings following a pre-pulse for left atrial myocytes isolated from mice infused with saline or Ang II for 3, 10 and 21 days. *p < 0.05 vs saline at indicated membrane potentials by two-way repeated measures ANOVA with a Holm-Sidak posthoc test. For right atrial myocytes *n*=12 for saline, 14 for Ang II (10 days) and 21 for Ang II (21 daysay). For left atrial myocytes *n*=11 for saline, 15 for Ang II (3 days), 17 for Ang II (10 days) and 12 for Ang II (21 days).

Next, the time course of the effects of Ang II on K^+^ currents was measured in right and left atrial myocytes. Initially, total I_K_ was measured between -120 and +80 mV using voltage clamp protocols with and without a pre-pulse to -40 mV to inactivate I_to_ (Figure 7A, Figure 8A). In right atrial myocytes, peak outward I_K_ was reduced at 21 days of Ang II infusion, but not at 10 days of Ang II infusion (Figures 7A,B). In left atrial myocytes, peak outward I_K_ was reduced to similar levels at 3, 10 and 21 days of Ang II infusion (Figures 7A,C). Similar patterns were observed after inactivation of I_to_ with a pre-pulse to -40 mV (Figure 8A). In these conditions, remaining peak outward I_K_ in right atrial myocytes was reduced only at 21 days of Ang II infusion (Figure 8B), while in left atrial myocytes remaining peak outward I_K_ was reduced similarly at all time points during Ang II infusion (Figure 8C).

**FIGURE 9:**
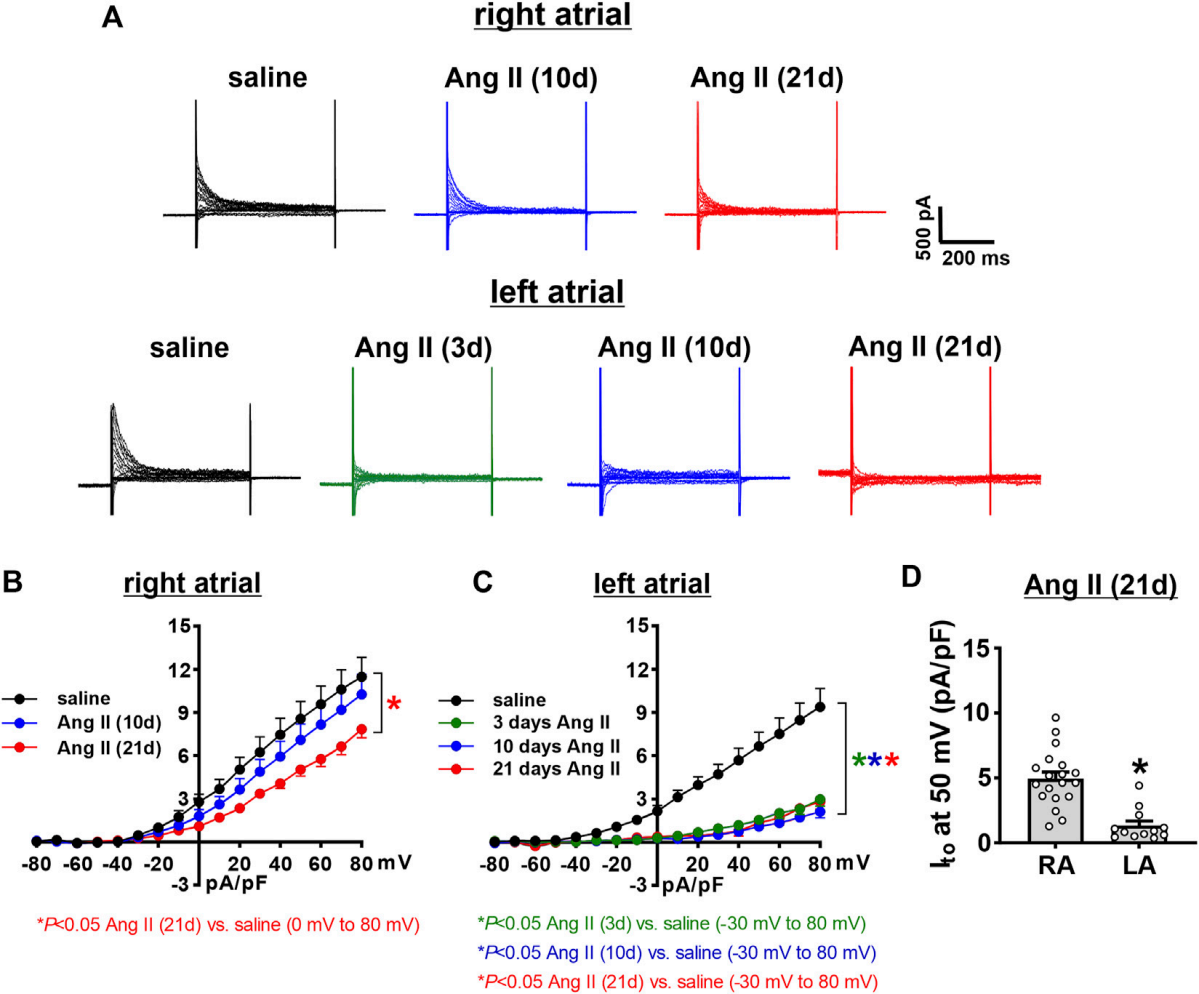
Time course of the effects of Ang II on the transient outward potassium current in right and left atrial myocytes. **(A)** Representative recordings showing the difference currents from I_K_ recordings with and without a pre-pulse (shown in Figure 6, Figure 7) in right and left atrial myocytes isolated from mice infused with saline for 21 days or Ang II for 3, 10, and 21 days. These difference currents represent I_to_ in each condition. **(B)** I_to_ IV curves in right atrial myocytes isolated from mice infused with saline or Ang II for 10 and 21 days. **(C)** I_to_ IV curves in left atrial myocytes isolated from mice infused with saline or Ang II for 3, 10 and 21 days. *p < 0.05 vs saline at indicated membrane potentials by two-way repeated measures ANOVA with a Holm-Sidak posthoc test. For right atrial myocytes *n*=12 for saline, 14 for Ang II (10 days) and 21 for Ang II (21 days). For left atrial myocytes *n*=11 for saline, 15 for Ang II (3 days), 17 for Ang II (10 days) and 12 for Ang II (21 days). **(D)** Comparison of I_to_ density at +50 mV in right atrial (RA) and left atrial (LA) myocytes after 21 days of Ang II infusion. *p < 0.05 vs RA by Student’s *t*-test; *n*=21 myocytes for RA and 12 myocytes for LA.

I_to_ was quantified as the difference current between I_K_ recordings with and without an inactivating pre-pulse (Figure 9A). In right atrial myocytes, I_to_ was reduced at 21 days of Ang II infusion, but not at 10 days of Ang II infusion (Figure 9B). I_to_ was reduced to comparable levels in left atrial myocytes at 3, 10 and 21 days of Ang II infusion (Figure 9C). After 21 days of Ang II infusion, I_to_ density was smaller in left atrial myocytes than right atrial myocytes (Figure 9D).

### Time course of Ang II effects on atrial fibrosis

**FIGURE 10:**
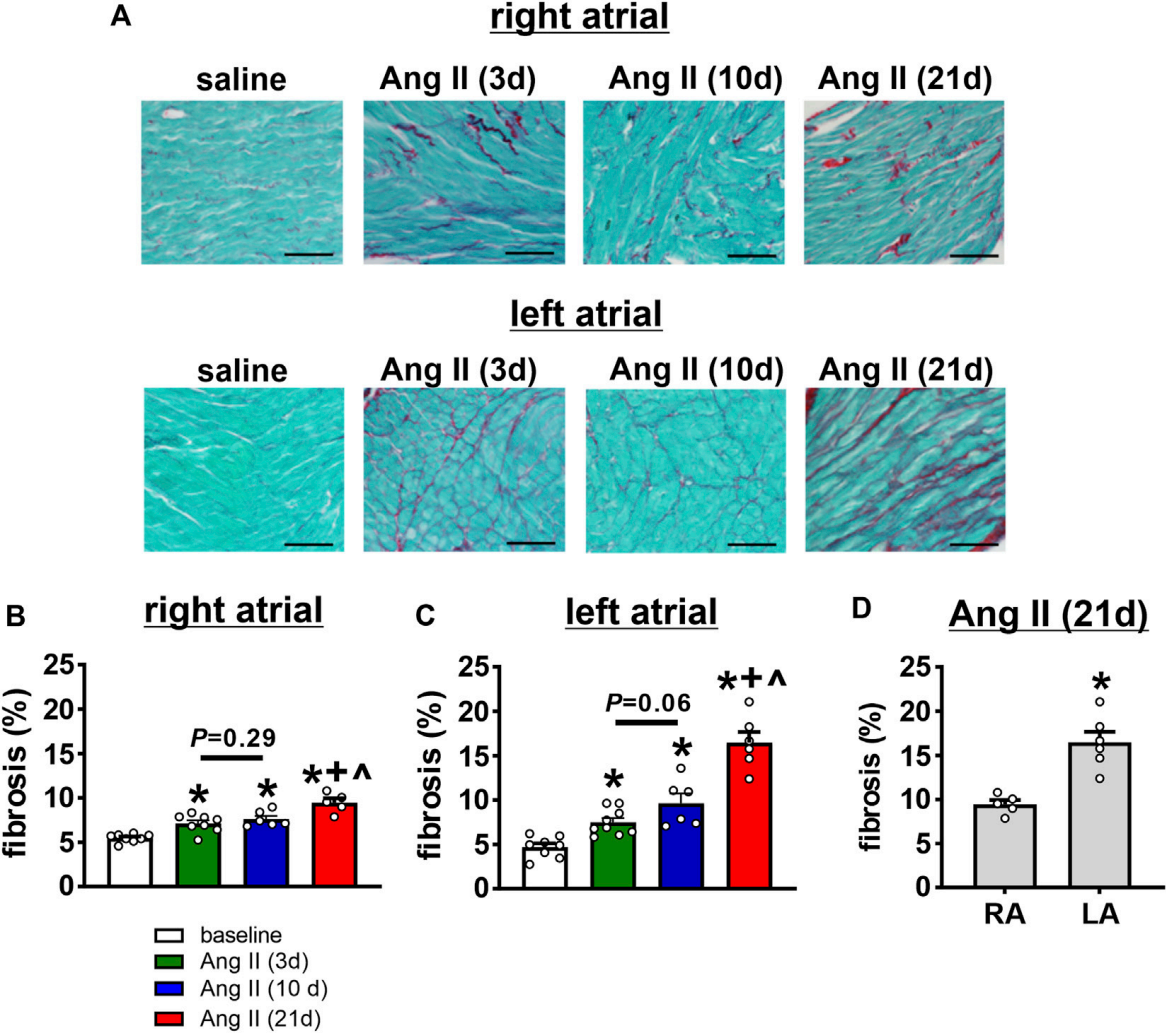
Time course of the effects of Ang II on right and left atrial fibrosis. **(A)** Representative images demonstrating interstitial fibrosis (collagen fibres stained red) in the right and left atria in mice infused with saline for 21 days or Ang II for 3, 10 and 21 days of Ang II. **(B–C)** Summary of interstitial fibrosis in the right **(B)** and left **(C)** atria of mice infused with saline or Ang II for 3, 10 and 21 days. *p < 0.05 vs saline, ^+^ *p* < 0.05 vs Ang II (3 days), ^^^ *p* < 0.05 vs Ang II (10 days) by one-way ANOVA with a Holm-Sidak posthoc test. For right atrium *n*=8 saline, 8 Ang II (3 days), 6 Ang II (10 days) and 5 Ang II (21 days). For left atrium *n*=8 for saline, 9 for Ang II (3 days), 6 for Ang II (10 days), 6 for Ang II (21 days). **(D)** Comparison of fibrosis in the right (RA) and left (LA) atria of mice after 21 days of Ang II infusion. *p < 0.05 vs RA by Student’s *t*-test; *n*=5 for RA and 6 for LA.

Disruptions in atrial conduction and increased susceptibility AF can also occur in association with structural remodeling of the atria due to fibrosis. Accordingly, the time course of the effects of Ang II on right and left atrial fibrosis was assessed (Figure 10A). Summary data demonstrate that right atrial fibrosis increased progressively from 3 to 21 days of Ang II infusion (Figure 10B). In the left atrium, fibrosis was also progressively increased at 3, 10, and 21 days of Ang II infusion (Figure 10C). Left atrial fibrosis after 21 days of Ang II infusion was greater than after 3 and 10 days of Ang II infusion (Figure 10C). Finally, following 21 days of Ang II infusion, left atrial fibrosis was greater than right atrial fibrosis (Figure 10D).

### Atrial expression of angiotensin II type 1 receptor

**FIGURE 11:**
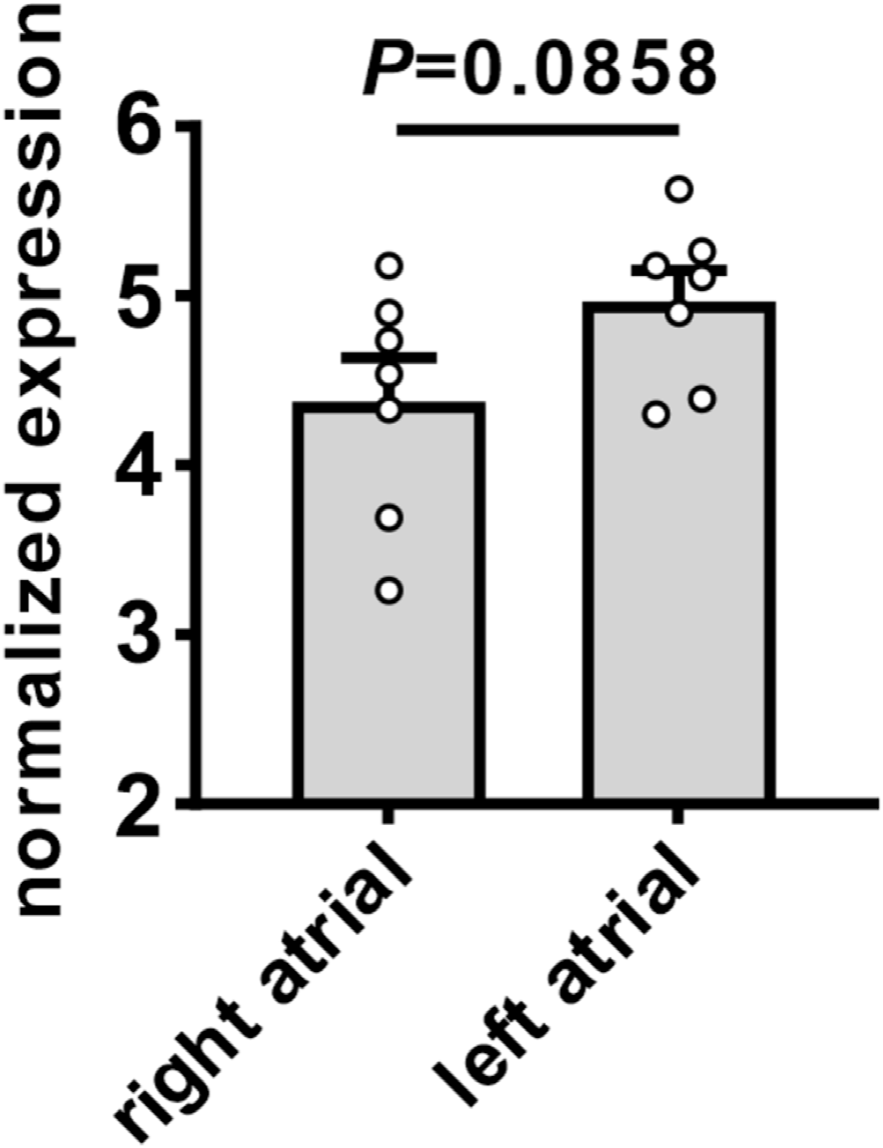
Expression of angiotensin II type 1 receptor in the right and left atria of mice. Comparison of mRNA expression of AT1R in the right and left atria of untreated wildtype mice. Data analyzed by Student’s *t*-test; *n*=7 for right atrial and 7 for left atrial groups.

AT1R mRNA expression was measured in the right and left atria of untreated wildtype mice (Figure 11). There was a trend towards a modest increase in AT1R mRNA expression in the left atrium compared to the right atrium.

## Discussion

This study examined the time course of the effects of Ang II on right and left atrial remodeling and how this impacts atrial electrophysiology and arrhythmogenesis. Ang II mediated hypertension has been previously shown to cause in increase in susceptibility to AF in association with electrical and structural remodeling in the atria (Purohit et al., 2013; Jansen et al., 2018; Jansen et al., 2019). However, the time course of these phenomena, how they progress specifically in the right and left atria, and how they associate with changes in atrial electrophysiology and susceptibility to AF is not well understood.

Ang II elicited an increase in systolic blood pressure within 3 days of initiation of Ang II infusion. Blood pressure reached a maximum by 10 days of Ang II infusion, showing no further increase at 21 days of Ang II. In association with these changes, atrial electrophysiology also began to change within 3 days of Ang II infusion. P wave duration showed progressive increases from 3 to 21 days of Ang II. Similarly, AERP was increased within 3 days of Ang II and further increased at 21 days of Ang II. Thus, while changes in systolic pressure and atrial electrophysiology (i.e. P wave duration, AERP) were evident within 3 days of Ang II infusion, these were progressive changes that became greater at 10 and 21 days of Ang II. Susceptibility to induced AF was not different at 3 days of Ang II, showed a clear trend towards being increased at 10 days of Ang II, and was statistically greater at 21 days of Ang II. This indicates that although changes in atrial electrophysiology began within 3 days of Ang II infusion, an increase in susceptibility to AF did not fully manifest until 10–21 days of Ang II suggesting that changes in atrial electrophysiology must progress to a threshold before an overt increase in AF susceptibility becomes evident.

Investigation of the changes in atrial AP morphology revealed important differences in the timing and extent of the effects of Ang II in right and left atrial myocytes. Right atrial myocytes displayed no changes in cell capacitance up to 21 days of Ang II infusion. Left atrial myocytes exhibited no changes in cell capacitance at 3 days of Ang II; however, there was a trend towards increased left atrial myocyte capacitance at 10 days of Ang II and a clear increase at 21 days of Ang II indicating the development of cellular hypertrophy in the left atrium only. Previously, it was shown that 21 days of Ang II infusion leads to a reduction in AP upstroke velocity in association with a reduction in atrial I_Na_ in the left atrium, but not the right atrium (Jansen et al., 2018; Jansen et al., 2019). The present study demonstrates that left atrial V_max_ is not different at 3 days of Ang II but is reduced by 10 days of Ang II infusion. In agreement with this, left atrial I_Na_ was reduced similarly at 10 and 21 days of Ang II, but not at 3 days of Ang II, in association with shifts in the voltage dependence of steady-state activation to more positive potentials. Right atrial V_max_ was not altered even after 21 days of Ang II and we have previously shown that I_Na_ is unaltered after 21 days of Ang II in right atrial myocytes (Jansen et al., 2018). The reduction in left atrial I_Na_ has been shown to occur due to increased protein kinase C (PKC) activity (Jansen et al., 2018; Jansen et al., 2019). This suggests that atrial PKC activity in Ang II infused mice does not become pathologically elevated in ways that affect I_Na_ until 10 days after Ang II infusion begins. These data also indicate that the reductions in left atrial I_Na_ and V_max_ contribute to the progressive increases in P wave duration (an indication of slowed atrial conduction), particularly from 10 days of Ang II infusion onwards. Although P wave duration was also prolonged at the earlier timepoint of 3 days of Ang II, this is not explained by changes in AP V_max_ or I_Na_. Based on the findings in this study, reductions in left atrial I_Na_ may play a central role the development of a substrate for AF since the reduction in I_Na_ coincided with the increase in susceptibility to AF with both becoming evident together at 10 days of Ang II infusion. This supports the hypothesis that a reduction in left atrial I_Na_ contributes importantly to reaching a threshold whereby changes in atrial electrophysiology (e.g. prolongation in P wave duration) result in increased susceptibility to pacing-induced AF.

Previously, it was shown that Ang II infusion for 21 days also leads to increases in atrial AP duration and that these increases are greater in the left atrium compared to the right atrium (Jansen et al., 2018). The present study now demonstrates that AP prolongation was evident even within 3 days of Ang II infusion and increased progressively up to 21 days of Ang II in left atrial myocytes. In contrast, AP prolongation was not evident in right atrial myocytes until the final time point studied (21 days of Ang II). AP durations were longer in left atrial myocytes than right atrial myocytes after 21 days of Ang II. Similar patterns were observed for changes in repolarizing I_K_, including I_to_, which was reduced at all time points in left atrial myocytes, but only at 21 days of Ang II in right atrial myocytes, with smaller I_to_ densities in left atrial myocytes compared to right atrial myocytes after 21 days of Ang II. These findings indicate that left atrial myocytes are susceptible to earlier and more extensive increases in AP duration during Ang II infusion while right atrial myocytes show smaller changes that take longer to develop. These findings are consistent with the increases in AERP observed *in vivo*.

Ang II mediated AF is also associated with the development of atrial fibrosis (Jansen et al., 2018; Jansen et al., 2019). Here it is demonstrated that right and left atrial fibrosis was evident as early as 3 days of Ang II infusion and further increased at 21 days of Ang II. At 21 days of Ang II infusion left atrial fibrosis was greater than right atrial fibrosis. These findings are consistent with data demonstrating that pro-fibrotic gene expression changes occur earlier in the left atrium compared to the right atrium (Jansen et al., 2019). Fibrosis is known to contribute to a slowing of atrial conduction due to the disruption of connections between myocytes (Nattel, 2017; Jansen et al., 2020; Aguilar et al., 2021). Thus, the earlier manifestation of atrial fibrosis at 3 days of Ang II could explain the increase in P wave duration observed at this time point, despite no differences in atrial I_Na_ at this time. As Ang II induced remodeling progresses, further increases in atrial fibrosis, along with the development of left atrial I_Na_ reductions, likely collectively contribute to progressive P wave prolongation and ultimately the increase in susceptibility to AF.

The basis for regional and temporal differences in the effects of Ang II on atrial structure and function are likely complex and could involve both direct and indirect effects on the atria. These could involve direct effects of Ang II on atrial myocytes and fibroblasts as well indirect effects on the atria secondary to the development of hypertension (Forrester et al., 2018; Jansen et al., 2020; Aguilar et al., 2021). Atrial stretch can contribute to atrial remodeling and the pathogenesis of AF (De Jong et al., 2011). Since the left atrium is under much higher pressure than the right atrium, this could contribute to more severe stretch-dependent electrical and structural remodeling observed in left vs right atrium. Consistent with this hypothesis, studies have shown that AT1R overexpression in the heart, which does not result in hypertension (Paradis et al., 2000), results in a less severe AF phenotype compared to chronic Ang II infusion (Jansen et al., 2018; Jansen et al., 2019; Demers et al., 2022). In addition, we observed a trend towards modestly higher AT1R expression in left atrial tissue compared to right atrial tissue. Whether this also contributes to more severe electrical and structural remodeling in the left atrium via direct effects of Ang II on atrial myocytes and fibroblasts is unknown. Additional studies will be required to investigate these factors and how they contribute to differences in temporal and regional atrial remodeling.

The occurrence of AF can be affected by the wavelength of re-entry, which is the product of the conduction velocity and the effective refractory period (Nattel, 2002; Jansen et al., 2020). We have previously shown that chronic Ang II infusion results substantial slowing of conduction in the atria in association with reduced atrial I_Na_ and the development of atrial fibrosis (Jansen et al., 2018; Jansen et al., 2019), which would favor re-entry and AF maintenance. While prolongation of AP duration in Ang II infused mice could increase the wavelength, which might reduce the likelihood of re-entry, increases in AP duration could also exacerbate conduction slowing, particularly at high heart rates, while also increasing the potential for conduction block (Burton and Cobbe, 2001; Carmeliet, 2019). Thus, it is possible that impaired conduction may be particularly important in creating a substrate for AF in Ang II infused mice. Furthermore, AP prolongation could increase the likelihood of early afterdepolarizations, which could trigger AF (Jansen et al., 2020). Additional studies will be required to determine the relative impacts of conduction impairments and AP prolongation on AF susceptibility.

## Conclusion

Electrical and structural remodeling are thought to be key determinants of AF susceptibility, including in hypertension; however, the regional and temporal patterns underlying atrial remodeling have not been well described. Here it is shown that remodeling in the left atrium develops earlier and progresses more extensively than in the right atrium. Our studies demonstrate that early, relatively modest levels of fibrosis and APD prolongation do lead to changes in atrial electrophysiology *in vivo* (increases in P wave duration and AERP), but by themselves do not result in substantial increases in AF susceptibility. Once these changes progress further, and reductions in left atrial AP V_max_ and I_Na_ are also manifested, the increase in susceptibility to AF becomes apparent. This indicates a critical role for the reduction in left atrial I_Na_, in combination with atrial fibrosis and AP prolongation, in AF development. These data provide new insight into the regional and temporal changes in atrial remodeling that create a substrate for AF in Ang II mediated hypertension.

## Data Availability

The original contributions presented in the study are included in the article/supplementary material, further inquiries can be directed to the corresponding author.
